# Sarcopenia and Related Functional Outcomes Following Hip Surgery Among Egyptian Geriatric Patients With Hip Fracture

**DOI:** 10.7759/cureus.43166

**Published:** 2023-08-08

**Authors:** Hoda T Sanad, Sarah A Hamza, Radwan G Metwaly, Hesham M Elbehery, Reem M. S ELbedewy

**Affiliations:** 1 Geriatrics, Ain Shams University, Cairo, EGY; 2 Orthopaedic Surgery, Ain Shams University, Cairo, EGY

**Keywords:** sarc-f questionnaire, orthogeriatrics, functional decline, sarcopenia, hip fracture

## Abstract

Background

Sarcopenia is highly prevalent among elderly patients with hip fracture. Studies reported a significant association between sarcopenia and clinical outcomes in patients with hip fractures. The current study aimed to determine the prevalence of sarcopenia among elderly patients with hip fracture and its effect on short-term functional outcomes, highlighting predictors of postoperative functional decline.

Methods

This is a cross-sectional study followed by a prospective cohort. Elderly patients (60 years and above) with hip fractures were recruited from the orthopedic department. Patients were followed by the ortho-geriatric team in the perioperative period and for three postoperative months. Patients were subjected to comprehensive geriatric assessment including a full history and physical examination. In the preoperative state and after three months of follow-up the following were assessed: functional independence using the Barthel index (BI); nutritional state using a checklist named DETERMINE Your Nutritional Health*;* sarcopenia using the SARC-F questionnaire assessing strength, ambulation, rising from a chair, climbing stairs, and fall history. Perioperative risk assessment and post-discharge care were obtained through medical records and by questioning patients or families. Preoperative sarcopenia was confirmed using the Ishii equation which is an equation that includes (age, calf circumference, and hand grip strength).

Results

Preoperative sarcopenia screening showed that 29.3% of patients suffered sarcopenia by SARC-F questionnaire and 28.6% by Ishii equation score. At the end of the follow-up, 57.9% of patients suffered sarcopenia by SARC-F questionnaire. There was a marked post-fracture decline in independence level; 52.1% had slight dependence in function, 27.1% had moderate dependence in function, and 20.7% had total dependence in function.

Conclusion

This study gives us the chance for a greater understanding of the negative effects of sarcopenia on the outcomes following hip fracture surgery in the geriatric population. It shows a prevalence of sarcopenia among the elderly with hip fractures at 29.3%. The elderly experience a marked post-fracture decline in their level of independence concerning basic activities of daily living. Those with older age, higher comorbidities, cognitive impairment, and functional dependence with poor nutritional state are more vulnerable to functional decline. Other perioperative risks include delayed surgery, surgery type, postoperative complications, longer hospital stays, lack of planned rehabilitative and nutritional plans, and postoperative depression. Early detection of sarcopenia helps establish early interventional plans to reverse such poor outcomes.

## Introduction

Sarcopenia is a common clinical condition among the elderly. It’s one of the geriatric syndromes with a multifactorial character and different biological mechanisms. It is characterized by progressive and generalized loss of skeletal muscle mass, strength, and function [1]. The European Working Group on Sarcopenia in Older People (EWGSOP2) updated its definition to depend mainly on low muscle strength as a key characteristic of sarcopenia; it uses low muscle quantity and quality to confirm sarcopenia diagnosis and identifies poor physical performance as indicative of severe sarcopenia [2].

Sarcopenia overlaps with frailty, which is characterized by fatigue, weight loss, impaired movement, and weakness and thus increases the risk for future fractures. Low muscle mass is positively related to lower bone mineral density (BMD) resulting in osteosarcopenia, which is a predictor of fracture. This is due to several chemokines, interleukins, and growth factors mediating the communication between bone and muscle [3].

Sarcopenia is highly prevalent among the elderly with hip fractures. The overall prevalence (for both sexes) of sarcopenia ranges from 11% to 76.4% depending on diagnostic tools [4]. The prevalence of sarcopenia in the acute period following a fracture varies widely, ranging from 12% to 95% in males and from 18% to 68% in females [5]. Hip fracture is a major public health problem mainly affecting the elderly, with high one-year mortality, considerable disability and dependence, and reduced quality of life [6]. Many studies reported a significant association between sarcopenia and clinical outcomes in patients with hip fractures. Those outcomes included mortality, length of hospital stay, discharge disposition, mobility, functional status, and quality of life. [7].

Multiple factors may precipitate postoperative sarcopenia, such as the impact of surgery, the overall physical and mental stress, reduced caloric intake during hospitalization, inflammation, medication, type of anesthesia, and medical comorbidities. Cumulative periods of disuse not only impact muscle loss during hospitalization but also contribute to impaired recovery and physical functioning after discharge [8].

Hospital-associated deconditioning contributes to the overall functional decline during and following periods of hospitalization, affecting long-term clinical outcomes, quality of life, the need for readmission, and permanent institutionalization. Greater attention to muscle weakness may promote better recovery after a hip fracture [4].

This study aimed to determine the prevalence of sarcopenia among the elderly with hip fractures who were admitted to a university hospital and its effect on short-term functional outcomes, highlighting predictors of postoperative functional decline.

## Materials and methods

This is a cross-sectional study followed by a prospective cohort. A total of 140 elderly (60 years and older) patients with proximal femur fractures were recruited from the orthopedic department. They were followed by the orthogeriatric team in the perioperative period and for three months after surgery. Ethical approval was obtained from the Research Ethics Committee of Ain Shams University Hospitals, Cairo, Egypt (approval no. FMASU M D378/2019). Written, informed consent was obtained from participants.

Exclusion criteria included refusal to participate in the study, previously non-ambulant patients, patients with dementia, patients with neuromuscular diseases, patients who were not surgically managed, patients with multiple fractures (either bilateral hip or associated with other fractures), and patients with hand problems impeding hand grip strength testing.

Patients were subjected to full history taking (from patients or relatives) and clinical assessment. Height was estimated through demi-span measurements [9]. Weight was estimated using predictive equations of actual body weight (ABW) [10]. Cognitive function was assessed using the abbreviated mental test (AMT) [11]. Mood was assessed using the Patient Health Questionnaire 2 (PHQ2) [12]. The functional state was assessed using the Barthel index (BI) [13]. And nutritional state was assessed using the DETERMINE Your Nutritional Health checklist [14].

Sarcopenia was screened preoperatively through the SARC-F questionnaire. The SARC-F is a recommended screening tool for sarcopenia by the EWGSOP2 [2]. It is a questionnaire that assesses five domains: strength, ambulation, rising from a chair, stair climbing, and history of falls. A score of four or more indicates a risk of sarcopenia [15]. Sarcopenia was further confirmed using the Ishii equation, including age, calf circumference, and hand grip strength testing. The equation cutoff for low muscle mass was less than 8.05 kg/m2 in males and less than 5.35 kg/m2 in females [16]. On the third-month follow-up, all individuals were reassessed for sarcopenia, function, nutrition, and depression using the same tools formerly mentioned.

Perioperative risk assessment was done through medical records, including the Charlson comorbidity index, the American Society of Anesthesiology (ASA) score, information about the type of fracture, time to surgery, type of surgery, type of anesthesia, postoperative complications, and length of hospital stay. Postdischarge care was assessed based on the presence or absence of involvement in formal rehabilitation and nutrition plans.

At the end of the follow-up, participants were classified according to postoperative functional decline as either stable or deteriorating in function using the BI score. Both groups were compared to detect predictors of such a decline.

Statistical analysis

The analysis of data was performed using SPSS Statistics version 16 (IBM Corp., Armonk, NY, USA). A description of all data in the form of the mean (M) and standard deviation (SD) for all quantitative variables was done. Frequency and percentage were calculated for all qualitative variables. Comparison between quantitative variables was done using the t-test to compare two groups. A comparison of qualitative variables was done using the chi-square test or Fisher’s exact test when appropriate. A significant level was measured at p≤0.05, and highly significant at p≤0.01.

## Results

For this study, 140 patients were recruited from the orthopedic surgery department from March 2021 to September 2021. The studied population involved 67 males and 73 females. Age ranged from 61.33 years to 75.29 years, with a mean of 6.98 years. Around 83.6% lived in urban regions, and 59.3% were educated. About 29.3% of them were current smokers. Body mass index (BMI) ranged from 22.98 Kg/m2 to 32.62 Kg/m2 with a mean of 4.82 Kg/m2. The number of comorbidities ranged from 1.13 to 3.45 with a mean of 1.16, while the Charlson comorbidity index ranged from 1.03 to 3.53 with a mean of 1.25 (Table 1).

**Table 1:** General characteristics of the studied population NOF: Neck of femur, CMN: Cephalomedullary nail, DHS: Dynamic hip screws, THA: Total hip arthroplasty, SD: Standard deviation, IQR: Interquartile range

Variables		N (140)
Age	Mean±SD	68.31 ± 6.98
Gender	Male	67 (47.9%)
	Female	73 (52.1%)
Charlson comorbidity index	Mean±SD	2.28 ± 1.25
Fracture type	NOF fracture	61 (43.6%)
	Sub trochanteric fracture	17 (12.1%)
	Intertrochanteric fracture	62 (44.3%)
Number of falls in the past year	Median (IQR)	1 (0 – 2)
Time to surgery	Less than or equal to 48 hours	94 (67.1%)
	More than 48 hours	46 (32.9%)
Type of surgery	CMN	67 (47.9%)
	DHS	21 (15.0%)
	Bipolar arthroplasty	36 (25.7%)
	THA	10 (7.1%)
	Cannulated Screws	6 (4.3%)
Postoperative delirium	Negative	108 (77.1%)
	Positive	32 (22.9%)
Postoperative infections	Negative	129 (92.1%)
	Positive	11 (7.9%)
Postoperative complications related to comorbidities	Negative	132 (94.3%)
	Positive	8 (5.7%)
Length of hospital stay in days	Mean±SD	6.90 ± 2.07
Postoperative rehabilitation	Not done	49 (35.0%)
	Done	91 (65.0%)
Postoperative nutritional plan	Not done	103 (73.6%)
	Done	37 (26.4%)

Around 90.0% had no family history of hip fracture, with a low prevalence of previous orthopedic surgical history (5.0%). Slippage resulted in 42.1% of the fractures, followed by high-energy trauma in 31.4%, while 26.4% had simple falls. Intertrochanteric fractures and the neck of the femur were the most common fractures seen in 44.3% and 43.6%, respectively. The rest were subtrochanteric fractures. The median of the past year's falls was 1 (0-2) (as seen in Table 1).

Regarding operative data, 67.1% of the studied population were operated on within 48 hours. Around 47.9% of them had cephalomedullary nails (CMN), 25.7% had bipolar arthroplasty, 15% had dynamic hip screws (DHS), 7.1% had total hip arthroplasty (THA), and 4.3% had cannulated screws. About 86.4% underwent spinal anesthesia; 22.9% of patients had postoperative delirium; 7.9% had postoperative infections (mainly chest infections); and 5.7% had postoperative comorbidity-related complications. Hospital stay was one week on average (6.90 ± 2.07 days) (as shown in Table 1). During the post-operative follow-up, 65.0% of the studied population underwent physiotherapy, while only 26.4% of them underwent nutritional plans. About 42.9% were screened positive for depression (Table 1).

The studied population was subjected to a comprehensive geriatric assessment of their preoperative condition; 85.7% were cognitively intact, and 14.3% had moderate cognitive abilities. Around 85.0% had a slight dependence in function, while 15.0% had a moderate dependence in function. About 23.6% suffered from depression, 74.3% had a good nutritional state, and 12.9% had a high nutritional risk. Preoperative sarcopenia screening showed that 29.3% of patients suffered from sarcopenia according to the SARC-F questionnaire and 28.6% according to the Ishii equation score.

At the end of the follow-up period, 52.1% had a slight dependence in function, 27.1% had a moderate dependence in function, and 20.7% had a total dependence in function. Around 67.9% had a good nutritional state, while 15.7% had a high nutritional risk. Sarcopenia screening showed that 57.9% of patients suffered from sarcopenia, per the SARC-F questionnaire (Table 2). Most of the studied population (85.0%) had no previous fracture, 11.4% had an old conservative fracture, and 3.6% had an old surgical fracture.

**Table 2:** Comparison of pre and postoperative assessment of function, nutrition, and sarcopenia among the studied population A p-value ≤0.05 is significant. A p-value ≤0.01 is highly significant. IQR: Interquartile range

Variables		Preoperative assessment	3rd-month postoperative follow-up	p-value
		N= 140	N= 140	
Barthel index (BI)	Full independence/Slight dependence	119 (85.0%)	73 (52.1%)	<0.001
	Moderate dependence	21 (15.0%)	38 (27.1%)	
	Total/Severe dependence	0 (0.0%)	29 (20.7%)	
DETERMINE nutrition checklist	Good	104 (74.3%)	95 (67.9%)	0.366
	Moderate risk	18 (12.9%)	23 (16.4%)	
	High risk	18 (12.9%)	22 (15.7%)	
SARC-F questionnaire	Sarcopenic	41 (29.3%)	81 (57.9%)	<0.001
	Non-sarcopenic	99 (70.7%)	59 (42.1%)	
SARC-F score	Median (IQR)	2 (1 – 4)	4 (1 – 7)	<0.001
	Range	0 – 8	1 – 10	

**Figure 1:**
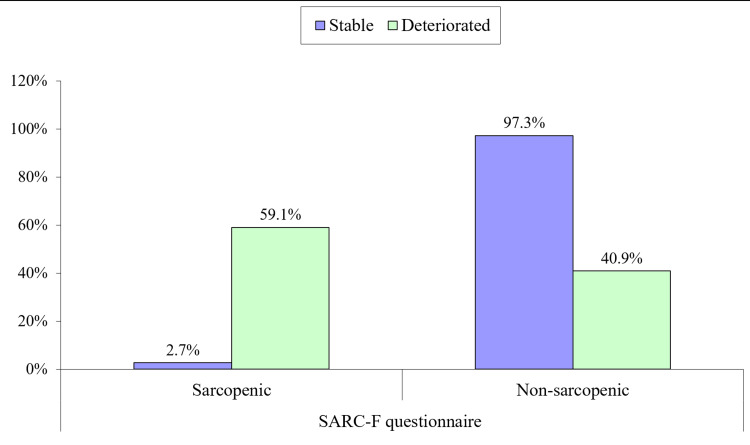
Preoperative sarcopenia (by SARC-F questionnaire) as a predictor of functional decline after three months

We aimed to figure out predictors of postoperative functional deterioration. Preoperative predictors of functional decline are shown in Table 3. Those who deteriorated in their function were older and had higher comorbidities. They had more cognitive and mood problems, moderate functional dependence, moderate to high nutritional risk, and were sarcopenic in their preoperative assessment via the SARC-F questionnaire (Figure 1). 

**Table 3:** Preoperative predictors of functional decline after the third-month follow-up A p-value ≤0.05 is significant. A p-value ≤0.01 is highly significant. ABT: Abbreviated mental test, BI: Barthel index, PHQ2: Patient health questionnaire, SD: Standard deviation

Variables		Functional decline after 3 months		p-value
		Stable	Deteriorated	
		No.= 74	No.= 66	
Age	Mean±SD	64.16 ± 4.55	72.95 ± 6.27	0.000
Gender	Male	43 (58.1%)	24 (36.4%)	0.010
	Female	31 (41.9%)	42 (63.6%)	
Charlson comorbidity index	Mean±SD	1.65 ± 0.94	2.98 ± 1.17	0.000
	Range	0 – 5	1 – 7	
Preoperative cognitive assessment by AMT	Normal	73 (98.6%)	47 (71.2%)	0.000
	Moderate	1 (1.4%)	19 (28.8%)	
	Severe	0 (0.0%)	0 (0.0%)	
Preoperative functional assessment by BI	Full independence/Slight dependence	73 (98.6%)	46 (69.7%)	0.000
	Moderate dependence	1 (1.4%)	20 (30.3%)	
	Total/severe dependence	0 (0.0%)	0 (0.0%)	
Preoperative mood assessment by PHQ2	Depressed	2 (2.7%)	31 (47.0%)	0.000
	Non-depressed	72 (97.3%)	35 (53.0%)	
Preoperative nutritional assessment by DETERMINE checklist	Good	73 (98.6%)	31 (47.0%)	0.000
	Moderate risk	0 (0.0%)	18 (27.3%)	
	High risk	1 (1.4%)	17 (25.8%)	
Preoperative sarcopenia assessment by SARC-F questionnaire	Sarcopenic	2 (2.7%)	39 (59.1%)	0.000
	Non-sarcopenic	72 (97.3%)	27 (40.9%)	

**Figure 2:**
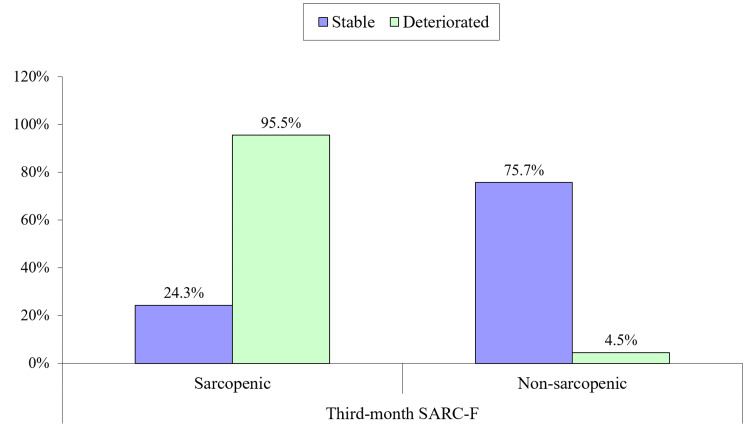
Sarcopenia at the third-month follow-up (assessed by SARC-F questionnaire) as a predictor of functional decline after three months

Postoperative predictors of functional decline are shown in Table 4. Those who deteriorated in their function had their operative intervention delayed. Dynamic hip screws and THA were associated with less functional decline. Those who deteriorated suffered more from postoperative delirium and other complications and had longer hospital stays. Those who deteriorated in function didn’t follow rehabilitation or nutritional plans and were sarcopenic, per the SARC-F questionnaire at the third-month follow-up (Figure 2). All the variables mentioned were statistically significant.

**Table 4:** Perioperative predictors of functional decline after three months of follow-up A p-value ≤0.05 is significant. A p-value ≤0.01 is highly significant. CMN: Cephalomedullary nail, DHS: Dynamic hip screws, THA: Total hip arthroplasty, SD: Standard deviation, IQR: Interquartile range

Variables		Functional decline after three months		p-value
		Stable	Deteriorated	
		No.= 74	No.= 66	
Time to surgery	Less than or equal to 48 hours	57 (77.0%)	37 (56.1%)	0.008
	More than 48 hours	17 (23.0%)	29 (43.9%)	
Type of surgery	CMN	34 (45.9%)	33 (50.0%)	0.001
	DHS	14 (18.9%)	7 (10.6%)	
	Bipolar arthroplasty	11 (14.9%)	25 (37.9%)	
	THA	9 (12.2%)	1 (1.5%)	
	Cannulated screws	6 (8.1%)	0 (0.0%)	
Postoperative delirium	Negative	73 (98.6%)	35 (53.0%)	0.000
	Positive	1 (1.4%)	31 (47.0%)	
Postoperative infections	Negative	72 (97.3%)	57 (86.4%)	0.016
	Positive	2 (2.7%)	9 (13.6%)	
Postoperative complications related to comorbidities	Negative	73 (98.6%)	59 (89.4%)	0.019
	Positive	1 (1.4%)	7 (10.6%)	
Length of hospital stay	Mean±SD	6.50 ±1.55	7.35 ±2.47	0.015
Postoperative rehabilitation	Not done	0 (0.0%)	49 (74.2%)	0.000
	Done	74 (100.0%)	17 (25.8%)	
Postoperative nutritional plan	Not done	39 (52.7%)	64 (97.0%)	0.000
	Done	35 (47.3%)	2 (3.0%)	
Postoperative depression	Not depressed	73 (98.6%)	7 (10.6%)	0.000
	Depressed	1 (1.4%)	59 (89.4%)	
Third-month SARC-F questionnaire	Sarcopenic	18 (24.3%)	63 (95.5%)	0.000
	Non-sarcopenic	56 (75.7%)	3 (4.5%)	

A logistic regression analysis was done to identify the most significant predictors of postoperative functional decline. Univariate logistic regression analysis showed that significant predictors include age above 67 years, female gender, Charlson comorbidity index of two or more, preoperative assessment (cognitive, functional, mood, nutrition, and sarcopenia), early surgery, use of bipolar arthroplasty, postoperative complications (delirium and infections), postoperative nutritional plans, postoperative depression, and postoperative sarcopenia. Multivariate logistic regression analysis showed that the most significant factors were age and postoperative depression (Table 5).

**Table 5:** Univariate and multivariate logistic regression analysis of the predictors of postoperative functional decline A p-value ≤0.05 is significant. A p-value ≤0.01 is highly significant. OR: Odds ratio, CI: Confidence interval, AMT: Abbreviated mental test, BI: Barthel index, PHQ2: Patient health questionnaire

Variables	Univariate				Multivariate			
	p-value	OR	95% CI for OR		p-value	OR	95% CI for OR	
			Lower	Upper			Lower	Upper
Age > 67	0.000	17.473	7.540	40.492	0.045	5.127	1.036	25.383
Female gender	0.011	2.427	1.228	4.800	-	-	-	-
Charlson comorbidity index > 2	0.000	9.184	4.214	20.012	-	-	-	-
Preoperative AMT	0.001	29.511	3.822	227.868	-	-	-	-
Preoperative BI	0.001	31.739	4.119	244.576	-	-	-	-
Preoperative PHQ2	0.000	31.886	7.216	140.894	-	-	-	-
Preoperative DETERMINE nutritional checklist	0.000	20.295	4.850	84.927	-	-	-	-
Preoperative SARC-F questionnaire	0.000	52.000	11.740	230.323	-	-	-	-
Time to surgery > 48 hours	0.009	2.628	1.269	5.441	-	-	-	-
Bipolar arthroplasty implant	0.003	3.492	1.552	7.857	0.054	5.225	0.973	28.072
Postoperative delirium	0.000	64.657	8.477	493.137	-	-	-	-
Postoperative infections	0.030	5.684	1.181	27.352	-	-	-	-
Postoperative complications related to comorbidities	0.117	3.437	0.735	16.086	-	-	-	-
Length of hospital stay > 6 days	0.051	1.973	0.998	3.900	-	-	-	-
Postoperative nutritional plan	0.000	28.718	6.540	126.097	-	-	-	-
Postoperative depression by PHQ2	0.000	615.286	73.618	5142.450	0.000	512.329	52.735	4977.339
Third-month SARC-F questionnaire	0.000	65.333	18.272	233.611	-	-	-	-

## Discussion

Sarcopenia is an important and highly prevalent health problem in the elderly that has a high rate of negative health-related outcomes, including falls and functional decline. Fractures result in significant impairment of independence for all basic and instrumental activities of daily living compared to baseline status. Functional outcomes following hip fractures are often unsatisfactory, and limited data about contributing factors exists [17].

Sarcopenia prevalence varies among studies according to diagnostic criteria, measurement tools, racial characteristics, the studied population, age groups, gender, dietary regimens, and quality of life. In this study, the prevalence of preoperative sarcopenia among participants was 29.3% when assessed by the SARC-F questionnaire and 28.6% by the Ishii equation. This is consistent with a systematic review, where the prevalence of sarcopenia ranged between 1% and 29% in community-dwelling populations [18]. In another systematic review, the estimated prevalence of sarcopenia ranged from 9.9% to 40.4% [19]. At the end of our follow-up, the prevalence of sarcopenia was recorded at 57.9% by the SARC-F questionnaire. Previous results stated that sarcopenia peaked two to six months after admission with a hip fracture [5].

We found preoperative sarcopenia to be statistically significant, with functional decline post-fracture. Around 97% of those who remained stable were non-sarcopenic in their pre-fracture state. While almost 60% of those who deteriorated were sarcopenic in their pre-fracture state. About 95.5% of those who deteriorated were sarcopenic in the third month of follow-up. In the literature, sarcopenia was found to be an important factor in poor functional outcomes following hip fracture surgery [20].

Some studies postulated that a poor recovery in sarcopenia status over time might be indicative of the weak effect of standard rehabilitation in the year following the fracture. It may also be the inability of such programs to overcome aging-related loss of muscle and strength as they focus more on eliminating disability in basic and instrumental activities of daily living than increasing muscle mass and strength [5]. More studies are needed to assess the response to specific interventions designed to increase muscle mass and strength.

During our follow-up period, a marked post-fracture decline in independence level in basic activities of daily living occurred. Around 85% of participants were fully independent or had a slight dependence pre-fracture. At the end of follow-up, almost 50% of participants suffered from moderate to severe dependence. Around 20.7% were not walking at all.

This finding goes with other studies, where walking recovery following hip fracture surgery was very poor at three to six months. Around 25% of participants failed to walk as well. Reasons behind this were pre-fracture walking abilities, the number of comorbidities, a longer hospital stay, and the lack of availability of rehabilitation [21].

Other studies state that recovery from a femoral fracture takes a long time. Patients were followed up for six months, and around 50% of patients recovered the pre-fracture level of activity of daily living, and only 25% recovered their instrumental activity of daily living [22].

According to our results, preoperative predictors of postoperative functional decline were related to preoperative sarcopenia, older age, a higher number of comorbidities, preoperative functional dependence, and a poor preoperative nutritional state. Multivariate logistic regression analysis showed that the most significant preoperative factor was age (>67 years). Higher age and comorbidities were associated with more decline in function at the end of the follow-up, which was similar to previous studies [7,23].

During our follow-up period, 98.6% of functionally stable participants had good nutritional status, while 53.1% of deteriorated participants had a moderate to high risk of malnutrition. Such results are similar to those found in other studies where pre-fracture nutritional status determined functional status at discharge. Participants who were malnourished or at risk of malnutrition before fracture had a lower functional status than those who were well-nourished [23,24]. Another study found that participants in the well-nourished group had a better BI score, up to three times that of the malnourished group, on discharge and at six months follow-up [25].

According to our results, perioperative predictors of postoperative functional decline were related to delayed surgery, type of surgery, postoperative complications, a longer hospital stay, and a lack of planned rehabilitative and nutritional plans. Early surgery was found to promote stability of function. Around 77% of those who remained stable in function had early surgery. Studies have reported that early surgical intervention is associated with better restoration of baseline function [26].

During our surgery, 48% had CMN, 25.7% had bipolar arthroplasty, 15% had DHS, 7% had THA, and 4.3% had cannulated screws. Choosing the implant depends on the type of fracture, pre-fracture activity level, joint congruence, presence of osteoarthritis, and available resources. The discrepancy in fracture type and stability guides the choice of surgery and implant, and accordingly, heterogeneity in rehabilitation programs may be the reason behind different functional outcomes within the follow-up period. We cannot recommend for or against a specific surgical intervention or implant choice from this study, and further patient stratification is needed. This can be one of the limitations of our study, although it is not one of the parameters for the primary outcome of the study.

The length of hospital stay was statistically correlated with functional deterioration. This goes with other studies where longer hospitalization without rehabilitation was found to extend bed rest and worsen walking status [21]. Therefore, the hospitalized elderly, especially during long periods, should have rehabilitative care.

Undergoing postoperative rehabilitative and nutritional plans affected functional outcomes. All participants who stayed stable in function were attached to formal rehabilitative plans. While 74% of those who deteriorated in function didn’t do the rehabilitation. Similarly, nutritional plans affected functional outcomes. Around 79% of those who deteriorated in function didn’t engage in nutritional interventions. While 47% of those who remained stable in function had undergone nutritional modifications.

Former studies state that interventions such as exercise and nutrition are effective in preventing and treating sarcopenia by preserving or even increasing muscle mass, strength, and physical performance. It is recommended to perform resistance training or multicomponent combined training for at least three months to have a significant impact on function [20,27]. Other studies showed that regular exercise improved muscle strength, physical performance, and quality of life [28]. Early implementation of physical activity during hospitalization played a major role in improving postoperative outcomes [8].

Despite having no evidence suggesting that high protein intake during short-term hospitalization decreases muscle loss, studies proved that lowering protein intake below habitual levels aggravates muscle loss during the immobilization period [8]. Studies also suggest that vitamin D may improve lower-limb muscle strength. Future research may reveal promising pharmacological agents for treating sarcopenia, such as myostatin antagonists, selective androgen receptor modulators, and skeletal troponin activators [27].

According to our results, cognitive abilities and mood directly affected functional outcomes. Participants with baseline moderate cognitive impairment and postoperative depression were associated with worsening BI scores through follow-up. Multivariate logistic regression analysis showed that the most significant postoperative factor was postoperative depression. Some other studies also related cognitive impairment and depression to postoperative functional recovery [29].

We experienced a complication rate of 36.5%. About 22.9% had postoperative delirium, 7.9% had postoperative chest or soft tissue infections, and 5.7% had comorbidity-related complications. Postoperative complications are one of the modifiable risks of postoperative functional decline [29]. Complication rates reported in the literature following hip fracture surgery vary widely between 12.5% and 57%. Such differences are due to differences in study design, study population, definition of a complication, assessment tools, and time of follow-up [30].

Limitations and strengths

This study is limited by its small sample size, and being a single-center study, it may not be representative of all geriatric populations with hip fractures in Egypt. There may be other modifiable factors that need further assessment. Details about socioeconomic factors, availability, and type of rehabilitation should have influenced our results. It would be even better to study each significant predictor on its own to avoid heterogeneity in the studied population and to create a better regression model of predictors of postoperative functional decline. Physical frailty is also an important predictor that affects the response of the geriatric population to acute stressors; therefore, it is essential to be assessed in future research. Nevertheless, our study opens the gate to further studies using such simple screening tools as the SARC-F and BI score for the assessment of sarcopenia and functional status, which can be done at outpatient clinics or using telemedicine.

## Conclusions

This study highlights the magnitude of sarcopenia and associated predictors of functional decline following hip fracture surgery in the geriatric population. The prevalence of sarcopenia among the elderly with hip fractures was 29.3% and reached 49.4% in the third month of postoperative follow-up.

Preoperative predictors include older age, higher comorbidities, cognitive impairment, functional dependence, and a poor nutritional state. Other perioperative predictors include delayed surgery, surgery type, postoperative complications, longer hospital stays, a lack of planned rehabilitative and nutritional plans, and postoperative depression. The early detection of such predictors helps establish early interventional plans to reverse such poor outcomes.
